# Electrocardiographic profile of South African Premier League football players: A descriptive cross-sectional study

**DOI:** 10.17159/2078-516X/2026/v38i1a22987

**Published:** 2026-09-15

**Authors:** L Maqina, TC Muluvhu, P Nongogo

**Affiliations:** 1 Department of Human Movement and Therapeutic Sciences, Faculty of Science, Tshwane University of Technology, Pretoria, South Africa; 2 Department of Tourism, Hospitality and Sport Management, Faculty of Management and Public Administration Sciences, Walter Sisulu University, East London, South Africa

**Keywords:** Athlete’s heart, Repolarisation, T-wave inversion, ST-elevation, J-point

## Abstract

**Background:**

High-intensity soccer participation induces benign structural and functional cardiac adaptations collectively known as the athlete’s heart. However, sudden cardiac death (SCD) remains an important concern, emphasising the need for appropriate cardiovascular screening among competitive soccer players, particularly in settings where routine pre-participation cardiac screening protocols are not consistently implemented across all levels of competition nationally.

**Objectives:**

To assess resting sport electrocardiogram (ECG) profiles of South African professional soccer players using international consensus standards for ECG interpretation in athletes.

**Methods:**

This cross-sectional study included 105 male South African Premier Soccer League players aged 17–39 years. Resting ECGs were recorded using the Custo-med Diagnostic Sport ECG device and interpreted according to international consensus criteria for athlete ECG interpretation.

**Results:**

Age and weight were not significantly associated with cardiac risk level (p=0.867 and p=0.400, respectively). Heart rate was significantly associated with risk level (p=0.002), with players presenting heart rates ≥76 bpm showing a higher proportion of moderate risk (63.6%). No significant associations were observed between age, weight, or heart rate and T-wave inversion. Heart rate was significantly associated with right atrial enlargement (p=0.037), particularly among players with heart rates ≥76 bpm, whereas age and weight showed no significant associations.

**Conclusion:**

Resting ECG screening may assist with the early identification of potentially important cardiac abnormalities in South African soccer players. Annual ECG screening should be considered for both development and senior squads, together with appropriate clinical follow-up and emergency preparedness. These findings support broader cardiovascular screening strategies aligned with international football medicine recommendations.

According to the Fédération Internationale de Football Association (FIFA) Big Count in 2006, there were nearly 265 million football players worldwide, with a gradual increase of approximately 10% every six years.^[1]^ Numerous physiological factors influence a football player’s performance at the elite level, including technical and biomechanical skills, tactical awareness, psychological attributes, and physiological capacity.^[2]^ In addition to endurance demands, players must execute numerous explosive movements, such as jumping, kicking, tackling, turning, sprinting, and altering pace.^[3]^ The high-intensity demands of football training lead to predictable, benign structural and functional cardiac changes in response to sustained physical activity. Collectively, these adaptations are referred to as the Athlete’s Heart.^[4]^

While such changes may be classified as pathological in nonexercising individuals, they are considered physiological and training-related in athletes and typically do not warrant further investigation. However, in some cases, further interpretation may be required to distinguish pathology from normal cases that do not warrant further investigations.^[5]^.

Research has increasingly emphasised the importance of clinicians involved in athlete screening being able to differentiate Electrocardiogram (ECG) abnormalities resulting from high-intensity physical training from those associated with increased cardiovascular risk for sudden cardiac arrest (SCA) or sudden cardiac death (SCD).^[6]^ While a preparticipation examination is recommended before competitive sports to identify such changes, there is substantial debate over the use of ECG to screen athletes for disorders associated with SCD, especially among Black African (BA) descent players, who have demonstrated higher rates of false-positive sports ECG findings during screening.^[6]^ In contrast, their Caucasian counterparts have demonstrated substantially lower false-positive rates. A false-positive result occurs when an ECG shows abnormalities that suggest an underlying heart condition, but the athlete is actually healthy with a structurally normal heart.^[7]^ Whether used to evaluate cardiovascular symptoms, family history of inherited cardiac disease, or premature SCD, or for routine screening of asymptomatic athletes, ECG interpretation is an essential skill for physicians involved in the cardiovascular care of athletes.^[8]^

According to data gathered by FIFA from football federations registered to report SCD) incidents, the epidemiology of SCD has been clarified within each confederation.^[1]^ The Union of European Football Associations (UEFA) reported the highest number of SCD events, accounting for 58% of all recorded cases.^[1]^ This predominance may be partly attributed to the enhanced administrative procedures implemented by European nations, which facilitate more accurate recording of SCD and SCA incidents.^[1]^.

The Confederation of African Football (CAF) reported the second-highest number of cases, with 20 incidents. This accounted for 14% of all recorded SCD cases, highlighting CAF as the second most affected federation among those monitored.^[1]^

Although SCA on the football field is infrequent, its occurrence carries a risk of death and substantial emotional impact on witnesses, warranting effective preventive and emergency response measures.^[9]^ Many of the previous cases that occurred during play may have been prevented with appropriate precautions, including prompt administration of cardiopulmonary resuscitation (CPR) and use of an automated external defibrillator (AED). Recent trends in South Africa have indicated a rise in cases of SCD amongst athletes. According to Slabbert and de Klerk (2012), this increase has highlighted a concerning gap in the documentation of pre-screening and treatment protocols for SCD. Despite the growing number of incidents, there remains limited information regarding the implementation of appropriate preventative measures and therapeutic interventions for affected individuals. ^[9]^ This highlights a critical gap in adherence to the FIFA Medical Assessment and Research Centre’s (F-MARC) recommendations for cardiac screening and emergency response in football.^[9]^ Subsequently, this study aimed to examine the ECG profiles of South African Premier Soccer League (PSL) players, assess resting ECGs of South African soccer players according to the international consensus standards for ECG interpretation in athletes, categorised by weight and determine rates of abnormal athlete’s heart among professional soccer players according to age and heart rate.

## Methods

### Study design

This study employed a descriptive cross-sectional design to assess the risk of SCD or SCA among South African professional football players. The study employed purposive sampling to recruit 105 professional male football players aged 16 to 40, who were registered with the South African PSL. Among these participants, 95 were BA and 10 were Caucasian. Only male football players were included in this study. This decision was based on the fact that there have been no documented cases of SCA or SCD among South African female professional football teams. The female teams have only recently achieved professional status; therefore, relevant cardiac events have not been reported in this population. Given the geographical and climatic variation across South Africa, the study focused on three professional teams: two inland and one coastal. Ethical approval for the study was obtained from the Faculty Committee for Research Ethics, Faculty of Science, Tshwane University of Technology (REC Ref #: REC2023-12-048 (SCI).

### Sampling procedure and participant recruitment

The Chief Medical Doctor of each team was emailed to explain the study and arrange a meeting. Out of 34 teams contacted, three participated. Team managers were then reached by email or phone and provided preseason schedules to ensure player availability. Before data collection, players attended an information session via PowerPoint covering the study, procedures, and facility needs. Medical staff received a separate briefing. Players were given information leaflets and consent forms before participating.

### Sample size

To recruit participants, the study utilised a purposive, non-probability sampling approach. Selection was based on specific population characteristics and aligned with the research objectives.^[10]^ The minimum number of participants required for the study was calculated using Slovin’s formula, based on the total of 562 registered PSL players. This formula indicated that at least 19 participants were needed to ensure reliable data. However, the study ultimately included 105 participants from the clubs that elected to participate. To ensure reliable data, a 0.05 margin of error was applied.

### Inclusion and exclusion criteria

The study included fully contracted players competing in the South African PSL aged 16–40 years. Participants were excluded if they had any existing cardiovascular or pulmonary conditions, as echocardiography was unavailable.

### Data collection

A 12-lead ECG assessment was performed using a CUSTO 300 device with sport ECG software. Athlete’s ECG findings were classified according to International Consensus Standards for ECG interpretation in athletes (Figure 1).

Electrode placement followed the 10 standard positions, in accordance with the ESC statement on ECG lead placement.^[11]^ The placement is as follows and can also be observed in (Figure 2): RA (Placed below the shoulder on the clavicle bone along the mid-axillary line), LA (Placed below the shoulder on the clavicle bone along the mid-axillary line), V1(Right) and V2 (left) sternal border on the fourth intercostal space.^[11]^ V3 can be placed midway between V2 and V4.^[11]^ V4, V5 and V6 are placed on a horizontal line on the first intercostal space, with V4 placed along the mid-clavicular line, V5 on the anterior-axillary line and V6 on the mid-axillary line just below the armpit.^[11]^ RL, which is sometimes referred to as the neutral (ground) electrode, can be placed on the right anterior superior iliac crest or just below the umbilicus along the anterior axillary line, and the LL electrode can be placed on the left anterior superior iliac crest or just below the umbilicus along the anterior axillary line.^[11]^

Figure 2 shows the parameters used to categorise players according to the ESC ECG categories. The following ECG changes were identified as common and training-related in professional football players: Sinus bradycardia, first-degree atrioventricular (AV) block, incomplete right bundle branch block (RBBB), early repolarisation, and isolated QRS voltage criteria for left ventricular hypertrophy (LVH). These findings were normal or indicative of a low risk for SCD.

Additional ECG findings—including left atrial enlargement, left axis deviation or left anterior hemiblock, right axis deviation or left posterior hemiblock, right ventricular hypertrophy, and complete left or right bundle branch block (LBBB or RBBB) occurring in isolation—were also classified as low risk. However, when any of these changes occurred two or more times within the ECG assessment, they were classified as borderline (moderate risk) or abnormal (high risk).

A single clinical primary researcher conducted all assessments throughout the study to ensure procedural consistency. Prior to the main study, a pilot study was conducted with a single second-division team (*n* = 14). This pilot study confirmed the reliability and feasibility of the data collection procedures, and no modifications to the protocol were required. All measurement equipment used in the study was calibrated on the day preceding testing. Calibration certificates were verified, and no corrective adjustments were necessary.

The collected data were entered into a Microsoft Excel spreadsheet and subsequently analysed using Stata Statistical Software, Release 19 (StataCorp. 2025, StataCorp LLC, College Station, TX, USA). Descriptive statistics, including means and standard deviations, were used to summarise continuous variables, while categorical variables and risk factors were presented as frequencies and percentages. Independent-samples t-tests were used to determine whether significant differences existed in the presence of risk factors according to Age, weight, and heart rate. Statistical significance was set at p ≤ 0.05.

## Results

### Descriptive Data (Mean, Standard Deviation, Min, Max)

Table 1 presents the descriptive statistics, including the mean, standard deviation (SD), and minimum and maximum values for age, Heart rate (HR), and weight among all players. The mean age of the players was 25.48 ± 4.8 years, with a minimum age of 17 years and a maximum of 39 years. The participants’ mean weight was 68.95 ± 9.48 kg, ranging from 52 kg to 97 kg. Additionally, the mean HR was 61.10 ± 11.25 bpm, with a minimum of 34 bpm and a maximum of 93 bpm.

### Association between demographic variables and risk level

Table 2 presents the demographic variables and the distribution of risk levels for 105 participants included in the analysis. There was no statistically significant association between age group and risk level (p = 0.867), nor between weight and risk level (p = 0.400). However, a statistically significant association was observed between heart rate and risk level (p = 0.002*), with participants in the highest heart rate category (≥76 bpm) demonstrating a higher proportion of moderate risk (63.6%).

### T-wave inversion

Table 3 presents the distribution of T-wave inversion (TWI) across age categories. There were no significant associations between age group, weight, or heart rate and the presence of T-wave inversions. In a 12-lead ECG, some TWI may indicate benign or pathological conditions, with repolarisation changes detected by alterations in the ST-segment or T-wave.^[12]^

### Pathological Q wave

Pathological Q waves were identified in 35 players (27%), whereas 70 players (54%) did not demonstrate pathological Q waves. Players with pathological Q waves were identified for further clinical evaluation to exclude underlying cardiac pathology.

### Right atrial enlargement (RAE)

Table 4 presents the distribution of right atrial enlargement (RAE) among all players (*N* = 105). This finding highlights a gap in the existing literature, as most previous studies have concentrated predominantly on the left heart chambers.^[13]^

No significant associations were observed between age group or weight and the presence of right atrial enlargement. However, heart rate was significantly associated with right atrial enlargement (*p* = 0.037), with a higher prevalence of RAE noted among participants whose heart rates were ≥76 bpm.

### Independent-Samples Kruskal-Wallis Test

Table 5 presents the results of the Independent-Samples Kruskal-Wallis test examining differences in age, weight, and heart rate (HR) across SCD risk categories. A statistically significant difference in heart rate distribution was observed across risk categories (*p* = 0.001). No statistically significant differences were found for age (*p* = 0.861) or weight (*p* = 0.113).

## Discussion

The study set examined the electrocardiographic characteristics of South African PSL players. Analysis of the risk distribution revealed that 21 players (20%) were classified as high risk, 67 (64%) as low risk, and 17 (16%) as moderate risk. The highest prevalence of moderate to high-risk classifications was observed among players aged 20–25 years. This finding is consistent with previous research, which reported that athletes aged 18–25 years accounted for the majority of SCA and SCD cases recorded between 1980 and 2007.^[14]^ Similarly, evidence from the FIFA sudden death registry demonstrated that young athletes in this age group frequently presented with ECG abnormalities.^[1]^ These observations raise concern regarding the possible presence of primary arrhythmia syndromes in younger athletes. Comparable trends have been identified in European cohorts and in other regions. A total of 37 players exhibited TWI, whereas 68 showed no evidence of TWI. In the general population, TWI is associated with cardiovascular risk factors and an increased risk of SCD.^[12]^ In athletes, however, the clinical significance of TWI depends on their distribution, with inverted T waves in certain leads (particularly outside the right precordial leads) warranting closer evaluation.^[12]^

The T-wave in an ECG represents the phase of rapid repolarisation of the ventricular action potential during a normal cardiac contraction.^[12]^ In a standard 12-lead ECG, certain TWI may indicate either benign or pathological conditions, particularly those related to repolarisation abnormalities, which can present as changes in the ST-segment or the T-wave itself.^[12]^ Primary repolarisation abnormalities can result from a variety of factors, including myocardial ischemia, structural heart disease, electrolyte imbalances, drug effects, alterations in sympathetic tone, and hyperventilation.^[12]^ For this reason, TWI are classified as an uncommon, training-related change in athletes, although in some cases they may indicate benign underlying conditions.^[12]^

Early ECG screening criteria were associated with high false-positive rates for TWI, resulting in unnecessary follow-up testing in athletes, particularly among BA players, who demonstrated a higher prevalence of ECG abnormalities compared to Caucasian athletes.^[7]^ More recent cohort studies reported a 6% prevalence of TWI in leads V1–V4 among BA without evidence of structural heart disease.^[15]^ Consequently, contemporary international ECG interpretation guidelines recognise TWI confined to V1–V4 as a possible physiological variant in BAs. Still, TWI in the lateral leads or those accompanied by ST-segment depressions warrant further evaluation.^[7]^

In the present study, the highest prevalence of TWI was observed in players aged 22–29 years (58%), supporting previous findings that younger football players may exhibit athlete’s heart adaptations in response to training demands.

Pathological Q waves were identified in 35 players (27%), while 70 (67%) showed no pathological Q waves. Pathological Q waves are indicative of transmural myocardial infarction (MI) and may indicate prior myocardial injury. In asymptomatic athletes, such findings require careful differential diagnosis to distinguish pathological causes from physiological adaptations. Based on these findings, 35 players require further diagnostic evaluation to exclude underlying structural or ischaemic pathology.

Among players with pathological Q waves, many also demonstrated marked sinus bradycardia (HRs between 40 and 55 bpm). This finding aligns with the international consensus, indicating that sinus bradycardia in athletes is often associated with structural and functional adaptations consistent with the athlete’s heart. Resting bradycardia (40–50 bpm) is common among football players and is generally considered a benign adaptation related to increased vagal tone and sinus node remodelling, specifically, downregulation of HCN4 channels. Although typically asymptomatic, it has been associated with an increased long-term risk of atrial fibrillation (AF) and, rarely, symptomatic bradycardia requiring pacing. In this cohort, most participants were considered to have normal cardiac profiles, while uncommon training-related changes were typically observed alongside other findings rather than in isolation. Accordingly, further assessment is required to exclude hypertrophic cardiomyopathy (HCM) or other structural pathology.

RAE was identified in 18 players (17%), while 87 (83%) showed no evidence of RAE. Existing literature predominantly focuses on left ventricular adaptations in athletes ^[1]^, with comparatively limited attention to right heart changes.^[13]^ While many studies describe left ventricular enlargement as the dominant adaptation, recent research indicates that intense exercise may also induce significant right heart morphological changes, which may carry clinical relevance. ^[13^

RAE, defined as abnormal enlargement of the right atrium, is typically associated with increased blood volume load or pressure and may result from underlying cardiac or pulmonary conditions. ^[13]^ Given the observed prevalence in this study, further investigation is warranted to determine whether these findings represent physiological adaptation or underlying pathology in this population.

There was no statistically significant association between age group and risk level (p = 0.867), nor between weight and risk level (p = 0.400). However, a statistically significant association was observed between heart rate and risk level (p = 0.002), with participants in the highest heart rate category (≥76 bpm) demonstrating a higher proportion of moderate risk (63.6%). There were no significant associations between age group, weight, or heart rate and the presence of T-wave inversions. Similarly, no significant associations were found between age group or weight and right atrial enlargement. However, heart rate was significantly associated with right atrial enlargement (p = 0.037), with a higher prevalence observed in participants with heart rates ≥76 bpm.

These findings support previous evidence that athletes with sinus bradycardia warrant further clinical evaluation. Furthermore, ethnicity has increasingly been recognised as an important determinant of ECG expression in athletes. BA exhibit a higher prevalence of repolarisation changes than other groups.^[1]^ Notably, approximately 90% of participants in this study were BA, which may partly explain the observed ECG patterns. Prior research has shown that BA frequently exhibit a repolarisation variant characterised by J-point elevation and convex ST-segment elevation in the anterior leads (V1–V4), followed by TWI.^[1]^ In the present study, however, most ECG changes were observed in the inferior and lateral leads rather than in V1–V4, and pathological Q waves were consistently noted in lead aVL among affected athletes. Some lateral ECG changes were also associated with features suggestive of HCM.

The findings from this study prompt consideration of whether South African football players possess a unique ECG profile compared to other athletic populations. This raises the question of whether current screening protocols are sufficient or whether more comprehensive measures are necessary to reliably exclude HCM and other forms of cardiac pathology in this group. It highlights the importance of tailored approaches to cardiovascular assessment to account for potential population-specific variations in ECG expression and associated risk factors. In recent years, South African football has witnessed a concerning rise in incidents of SCA and SCD among players. Historically, the country had not reported any such cases until a series of high-profile events brought the issue into the national spotlight. The first widely recognised case occurred in 2021 when George Maluleka experienced an SCA during a PSL match, an event that was broadcast live and shocked viewers across the nation. This incident marked a turning point, highlighting the urgency of addressing cardiac health in the sport. Shortly after the Maluleka case, the football community was again shaken by the sudden death of Siphamandla Mtolo, who collapsed during a training session. The sequence of events did not end there. Sinamandla Zondi collapsed during his warm-up before a match between Durban City and Milford. He was rushed to the hospital but tragically passed away en route.

Following the detection of a significant proportion of abnormal findings during the SCD screening process in this study, these results were formally communicated to the relevant football teams. Based on these outcomes, it was recommended that enhanced cardiovascular screening protocols be integrated into the pre-season medical assessments for players. This proposed initiative aims to improve the early detection and management of cardiac abnormalities. Furthermore, these enhanced protocols are planned to be part of an upcoming longitudinal study that will not only incorporate comprehensive echocardiographic evaluations but also ensure the involvement of a cardiologist in the assessment process. This multidisciplinary approach is intended to provide a more robust evaluation of cardiovascular risk and improve clinical outcomes for South African football players. In the interim period, sports physicians were instructed to pursue further differential diagnoses for affected soccer players. The physician’s responsibility to carry out this follow-up assessment was outlined in the informed consent provided to participants, ensuring ethical management and continuity of care.

### Limitations of the study

Several limitations should be considered when interpreting the findings. First, team participation was limited. Of the 34 clubs approached, only three teams agreed to participate. Although efforts were made to engage professional bodies to facilitate recruitment within the South African professional league, the low response rate may limit the generalisability of the findings. The sample may not represent the full diversity of playing levels, training regimens, or ethnic backgrounds across the country.

Second, an echocardiographic (ECHO) assessment was not performed. The study relied solely on ECG findings without confirmatory imaging to validate abnormalities identified during screening. Future studies should adopt a multidisciplinary approach that integrates ECG and ECHO assessments to enhance diagnostic accuracy, including collaboration with cardiologists.

Third, the cross-sectional design captures data at a single point in time, limiting the ability to establish causal relationships between ECG findings and SCD risk or to predict long-term cardiovascular outcomes. Longitudinal or interventional designs would provide greater insight into the progression of ECG changes over an athlete’s playing career and clarify whether identified abnormalities translate into adverse clinical outcomes.

Fourth, the absence of a non-athlete control group limited the ability to distinguish athlete-specific cardiac adaptations from general population variations. Comparison with age-matched sedentary individuals would strengthen the interpretation of the observed ECG changes.

Fifth, risk categorisation was based solely on ECG findings without clinical correlation or family history, which may have affected the accuracy of risk stratification. Comprehensive screening, including personal and family medical history, would provide a more robust risk assessment. In addition, follow-up clinical evaluation of high-risk athletes was limited, restricting assessment of subsequent outcomes.

Finally, although the use of a single assessor ensured procedural consistency, it may have introduced measurement bias and limited inter-observer validation.

## Conclusion

The ECG profiles observed among South African football players indicate a notable proportion of individuals categorised as moderate-to-high risk for sudden cardiac arrest SCA and SCD. Notably, the patterns identified in the presentation of the athlete’s heart within this group differ from those typically seen in other athletic populations. These variations underscore the importance of recognising population-specific ECG expressions, which may influence both risk assessment and clinical management strategies.

To address these challenges, there is a clear need for further comprehensive investigation to establish ECG standards tailored to the South African athlete population. Such standards would help to improve both the accuracy and reproducibility of ECG interpretation, ensuring that the screening process is fit for purpose. The value of systematic evaluation using standardised criteria has been demonstrated in previous studies, which show that this approach significantly enhances diagnostic accuracy in athletes.

Consequently, future research should focus on developing reliable, context-specific diagnostic protocols for South African football leagues. These protocols would enable physicians from various disciplines to assess athletes more accurately during cardiovascular screening, thereby supporting early detection and management of cardiac abnormalities. By advancing population-specific standards and systematic evaluation methods, the effectiveness of screening programmes can be optimised for the unique needs of South African footballers.

## Figures and Tables

**Fig. 1:**
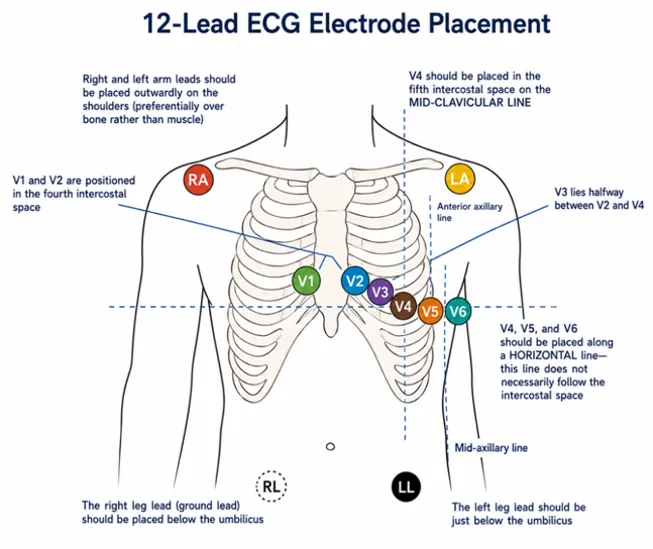
12-Lead ECG electrode placement. ECG, electrocardiogram; RA, right arm; LA, left arm; RL, right leg; LL, left leg. Figure adopted from Fletcher et al. (2001).

**Fig. 2:**
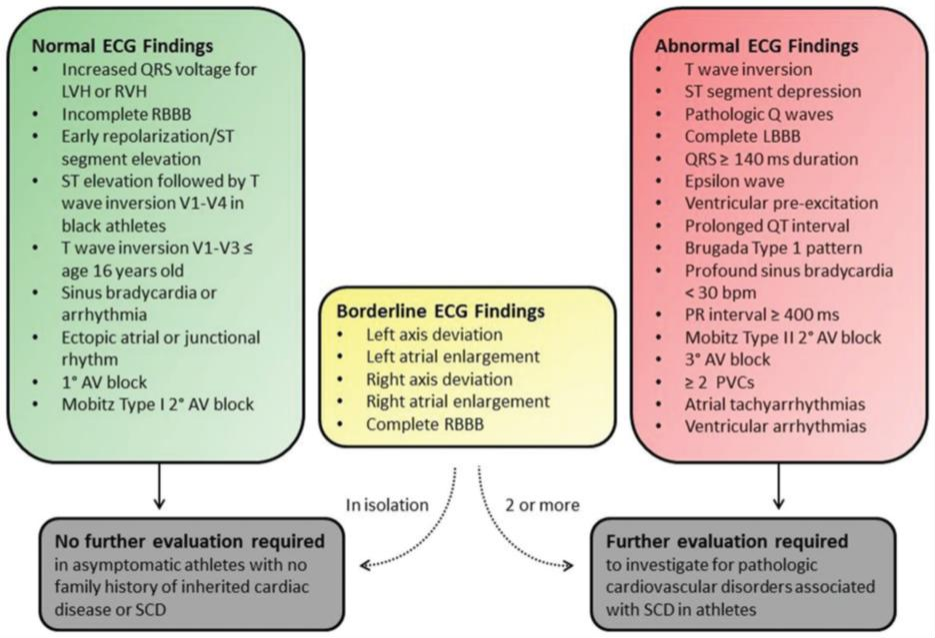
International consensus standards for ECG interpretation in athletes. ECG, electrocardiogram; RBBB, right bundle branch block; AV, atrioventricular; LBBB, left bundle branch block. Figure adopted from Drezner (2012).

**Table 1:** Descriptive data of participants (n = 105)

Variables	Mean ± SD	Minimum	Maximum
Age (years)	26 ± 9	17	9
Hr (bpm)	61 ± 11	34	3
Weight (kg)	69.0 ± 9.5	52.0	7.0

**Table 2:** Association between demographic variables and risk level (n = 105)

Variable	Category	High n (%)	Low n (%)	Moderate n (%)	Total n	P-value
**Age group (years)**	17–21	4 (16.0)	17 (68.0)	4 (16.0)	25	0.867
	22–29	13 (22.4)	37 (63.8)	8 (13.8)	58	
	≥30	4 (18.2)	13 (59.1)	5 (22.7)	22	

**Weight (kg)**	<65	11 (25.0)	28 (63.6)	5 (11.4)	44	0.400
	66–75	8 (20.5)	25 (64.1)	6 (15.4)	39	
	≥76	2 (9.1)	14 (63.6)	6 (27.3)	22	

**Heart rate (bpm)**	<60	14 (25.0)	37 (66.1)	5 (8.9)	56	0.002*
	60–75	7 (18.4)	26 (68.4)	5 (13.2)	38	
	≥76	0 (0.0)	4 (36.4)	7 (63.6)	11	

* indicates significance at p<0.05.

**Table 3:** Association between variables and T-wave inversions (n = 105)

Variable	Category	No n (%)	Yes n (%)	Total (n)	P-value
**Age group (years)**	17–21	14 (56.0)	11 (44.0)	25	0.480
	22–29	38 (65.5)	20 (34.5)	58	
	≥30	16 (72.7)	6 (27.3)	22	

**Weight (kg)**	<65	24 (54.6)	20 (45.5)	44	0.090
	66–75	26 (66.7)	13 (33.3)	39	
	≥76	18 (81.8)	4 (18.2)	22	

**Heart rate (bpm)**	<60	34 (60.7)	22 (39.3)	56	0.441
	60–75	25 (65.8)	13 (34.2)	38	
	≥76	9 (81.8)	2 (18.2)	11	

No, no T-wave inversions present; Yes, T-wave inversions were present.

**Table 4:** Association between variables and right atrial enlargement (n = 105)

Variable	Category	No n (%)	Yes n (%)	Total (n)	P-value
**Age group (years)**	17–21	21 (84.0)	4 (16.0)	25	0.732
	22–29	49 (84.5)	9 (15.5)	58	
	≥30	17 (77.3)	5 (22.7)	22	

**Weight (kg)**	<65	36 (81.8)	8 (18.2)	44	0.946
	66–75	33 (84.6)	6 (15.4)	39	
	≥76	18 (81.8)	4 (18.2)	22	

**Heart rate (bpm)**	<60	47 (83.9)	9 (16.1)	56	**0.037** *
	60–75	34 (89.5)	4 (10.5)	38	
	≥76	6 (54.6)	5 (45.5)	11	

* indicates significance at p<0.05.

No, no enlargement present; Yes, enlargement was present.

**Table 5:** Independent-Samples Kruskal-Wallis test results

Null hypothesis	Significance (*p* value)	Decision

The age distribution is the same across SCD risk categories.	0.861	Retain the null hypothesis.
The distribution of weight is the same across SCD risk categories.	0.113	Retain the null hypothesis.
The distribution of HR is the same across SCD risk categories.	0.001*	Reject the null hypothesis.

* indicates significance at p<0.05.
